# Photoanthropometric craniofacial parameters in individuals with osteogenesis imperfecta

**DOI:** 10.4317/medoral.26083

**Published:** 2023-10-12

**Authors:** Felipe Franco Marçal, Luiza Lassi de Araújo Lopes, Fábio Wildson Gurgel Costa, Lara Matos Moreno, Jorge Luiz Moreira Freire Júnior, Cauby Maia Chaves Júnior, Paulo Goberlânio de Barros Silva, Erlane Marques Ribeiro, Cristiane Sá Roriz Fonteles, Thyciana Rodrigues Ribeiro

**Affiliations:** 1 PhD, Postgraduate Program in Dentistry, Federal University of Ceará, Fortaleza, Ceará, Brazil; 2 PhD, Adjunct Professor, Department of Clinical Dentistry, Federal University of Ceará, Fortaleza, Brazil; 3 DDS, Graduate student, Federal University of Ceará, Fortaleza, Ceará, Brazil; 4 PhD, Professor, School of Dentistry, Christus University Center, Fortaleza, Brazil; 5 PhD, Professor, School of Medicine, Christus University Center, Fortaleza, Brazil; 6 PhD, Division of Genetics, Children’s Hospital Albert Sabin, Fortaleza, Brazil; 7 PhD, Titular Professor, Department of Clinical Dentistry, Federal University of Ceará, Fortaleza, Brazil

## Abstract

**Background:**

This study aimed to evaluate facial photoanthropometric parameters in patients with OI.

**Material and Methods:**

We selected 20 Brazilian patients diagnosed with OI treated at the Extension Service for Minors in Need of Specialized Treatment of the Dentistry Course at the Federal University of Ceará (Fortaleza, Brazil), of both sexes, without age restriction, and able to understand and sign the informed consent form (ICF). As a control group, 38 non-syndromic Brazilian individuals, categorized as ASA I, able to understand and sign the ICF, matched by sex, age, and Legan and Burstone facial profile were selected. The exclusion criteria were: previous orthodontic treatment, craniofacial trauma and/or surgery, and the presence of any other systemic diseases. Photoanthropometric analysis of the 18 facial parameters proposed by Stengel-Rutkowski *et al*. (1984), previously established in the literature for craniofacial syndromes, were conducted. A single examiner digitally performed all effective and angular measurements with the CorelDRAWX7® software.

**Results:**

Horizontally shortened ears (*p*<0.001) but larger in height in relation to the face (*p*=0.012) were shown to be alterations belonging to individuals with OI.

**Conclusions:**

OI patients present distinct photoanthropometric parameters inherent in this condition.

** Key words:**Osteogenesis imperfecta, face, photography.

## Introduction

Osteogenesis imperfecta (OI) encompasses a group of heterogeneous hereditary connective tissue syndromes, mainly characterized by bone fragility, which leads to frequent fractures and the development of disabling bone deformities (1,2). Most OI patients present dominant mutations in either COL1A1 or COL1A2 genes, which encode type I collagen (3). This syndrome is considered the most common genetic bone disease, affecting 1 in 10,000 individuals across all ethnic groups (4).

In most cases, OI is a result of autosomal dominant mutations that cause primary defects in type I collagen production. The remaining cases may arise pathogenic variations in genes of non-collagen-producing cells, encoding proteins involved in collagen biosynthesis, or transcription factors and signaling molecules related to bone cell differentiation and mineralization, which are most commonly associated with an autosomal recessive inheritance (5). Type I collagen is the main structural constituent of bone and dentin; thus, OI mutations in this protein often lead to quantitative and qualitative changes, resulting in reduced bone tissue mineralization (3). In a previous study with Brazilian patients with OI, we found a significant prevalence of dental alterations, notably dentinogenesis imperfecta, which occurred in 75% of cases (6).

Phenotype variability underlines the complexity in understanding the etiopathogenesis of these alterations. In addition to bone fragility, which increases susceptibility to multiple fractures, patients may exhibit short stature, hearing loss, blue sclera, and type I dentinogenesis imperfect (3). Based on the effects on these multiple genetic, clinical, and radiographic parameters, a classification into four subtypes was created to identify the most common OI variations reported in the scientific literature, thereby enabling a better description and analysis of OI and its different phenotypic repercussions (7).

Therefore, greater attention has been given to the various changes observed in this syndrome, including craniofacial alterations. Abnormal craniofacial development may cause functional impairment in speech and mastication, in addition to aesthetic problems (8,9). The documented clinical craniofacial findings of this syndrome were focused on the triangular shape of the face, relatively large head size, and soft calvaria (3).

Although the diagnostic criteria for OI have been already established in the literature from a genetic and clinical point of view, there is still a paucity of anthropometric data on phenotypic traits in patients with this rare condition. In this context, the aim of the present study was to evaluate photoanthropometric craniofacial parameters in Brazilian individuals with OI to better describe and characterize its craniofacial aspects.

## Material and Methods

- Study design, ethical aspects, and participants

This cross-sectional observational study was approved by the human research ethics committees of the Federal University of Ceará (UFC) (approval number #1,234,669) and conform to STROBE Guidelines. The convenience sample of this study comprised 20 participants with a medical diagnosis of OI (OI [case] group) referred to the dental care service for patients with special needs (Extension Service for Minors in Need of Specialized Treatment) of the Dentistry course at the UFC (Fortaleza, Brazil). Most patients diagnosed with OI came from a Brazilian referral center for rare diseases (Albert Sabin Children’s Hospital). Thirty-eight volunteers without OI (control group), referred from the Pediatric Dentistry Clinic at the UFC, were also recruited and matched by sex, age, and facial profile according to Legan and Burstone’s soft tissue cephalometric analysis (10).

- Eligibility Criteria

Inclusion criteria for the OI group were a) previous medical diagnosis of OI; b) born in Brazil; c) no sex or age restriction; d) individuals capable of understanding and signing the informed consent form (ICF), or, in case of minors, whose parents or legal guardians signed the ICF agreeing to participate in the study. Participants of the control group were non-syndromic volunteers, born in Brazil, without systemic comorbidities (ASA I - American Association of Anesthesiologists), matched by sex, age, and Legan and Burstone analysis, and who consented to participate in the study following ethical precepts. For age matching of controls, a margin of ±2 years was assumed for patients aged 15-30, and ±5 years for patients aged 30-45 years.

For both groups (case and control), the following exclusion criteria were considered: a) previous malocclusion treatment; b) history of previous craniofacial trauma and/or surgery; c) diagnosis of systemic diseases other than OI for the case group.

- Photoanthropometric analysis

The photographic images were taken with a Nikon D3100 DSLR camera, 55 mm lens, standardized in *P* conFiguration, ISO100, with flash, and Daylight mode on. For the standardization of the reference planes, each patient remained comfortably seated with their head positioned in a natural vertical position. With the interpupillary line parallel to the ground and the patient staring at him/herself through a mirror positioned at head height, frontal and lateral views were taken. A cotton string was fixed to the ceiling with a weight at its end to be used as a vertical position reference. The photographs were taken with a standard distance of 1.5 meters from the participant.

**Figure 1:**
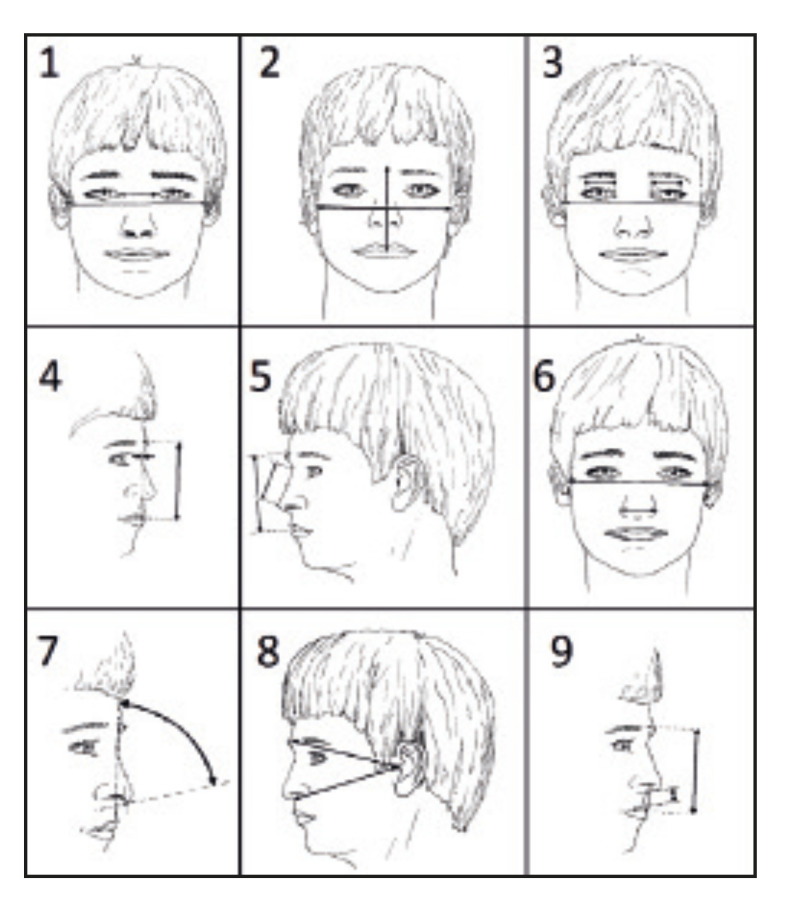
Representative images of facial parameters 1-9 based on Stengel-Rutkowski *et al*., 1984. 1) Intercanthal distance [Inner Intercanthal Distance (ICD)/Horizontal Facial Reference (HFR)]. 2) Midfacial height [Midfacial Height (MFH)/Horizontal Facial Reference (HFR)]. 3) Width of the palpebral fissures [Width of the Palpebral Fissures (PFW)/Horizontal Facial Reference (HFR)]. 4) Nasal root depth [Nasal Root Depth (NRD)/Vertical Facial Reference (RVF)]. 5) Posterior nasal length [Posterior Nasal Length (PNL)/Vertical Facial Reference (RVF)]. 6) Interalar distance [Interalar Distance (IAD)/Horizontal Facial Reference (HFR)]. 7) Inclination of the nasal base. 8) Prominence of the maxilla. 9) Nasolabial distance [Nasolabial distance (NLD)/Vertical Facial Reference (RVF)].

**Figure 2:**
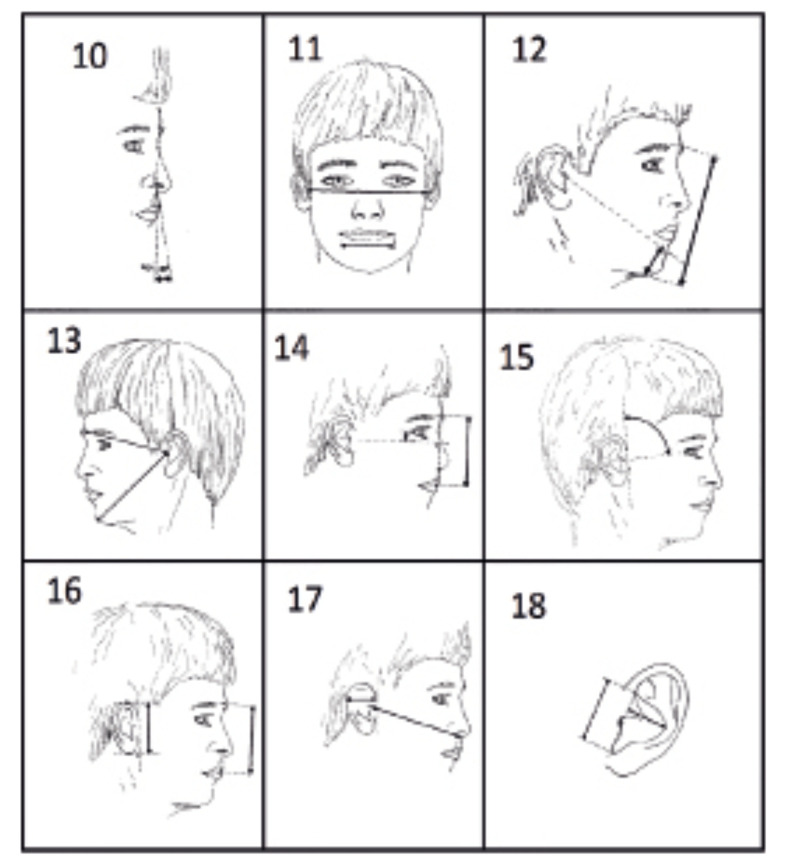
Representative images of facial parameters 10-18 by Stengel-Rutkowski *et al*., 1984. 10) Integumental Upper Lip. 11) Mouth width [Mouth Width (MW)/Horizontal Facial Reference (HFR)]. 12) Chin height [Chin Height (CH)/Total Facial Height (TFH)]. 13) Prominence of the chin. 14) Position of the ears [Vertical Position of the Ears (VPE)/Vertical Facial Reference (RVF)]. 15) Inclination of the ear insertion line. 16) Ear length [Vertical Length of the Ears (VLE)/Vertical Facial Reference (RVF)]. 17) Ear width [Ear width (EW)/Maxillary length (ML)]. 18) Conchae width [Conchae Width (CW)/Conchae Length (CL)].

For the photoanthropometric analysis, we adopted the18 craniofacial indices established by Stengel-Rutkowski *et al*. (1984) (11) (Fig. 1, Fig. 2), which comprises 5 angular measurements and 13 percentage distributions, i.e., proportions of individual linear measurements in relation to a facial reference. The individual measurements of the 13 parameters of percentage distributions were described and analyzed according to the nomenclatures in Fig. 1 and Fig. 2.

A previously trained examiner performed all facial analyses with the imaging software CorelDRAW X7®, in which the points were established by the operator in the virtual system, and the software performed all linear or angular measurements.

- Statistical analysis

Data were submitted to statistical analysis, using the Statistical Package for the Social Sciences (SPSS) software, version 20.0, in Windows® environment. Data were expressed as mean and standard deviation values, submitted to the Kolmogorov-Smirnov normality test, and compared using Student's t-test. Additionally, ROC curves were constructed to calculate the estimated diagnostic cutoff points of the cases. The area under the curve, sensitivity, and specificity values of the cutoff points were also calculated. The level of statistical significance adopted for all tests was 5% (*p* < 0.05).

## Results

This study comprised 20 patients with OI (8 males and 12 females) and 38 patients without OI (15 males and 23 females). The mean age of the patients was 15.24 years.

Most individual distances of the photoanthropometric indices were statistically reduced in patients with OI (Table 1): inner intercanthal distance (mm) (*p*<0.001), horizontal facial reference (mm) (*p*<0.001), midfacial height mean (mm) (*p*<0.001), width of palpebral fissures (mm) (*p*=0.009), nasal root depth (mm) (*p*=0.044), interalar distance (mm) (*p*=0.001) , mouth width (*p*<0.001), total facial height (mm) (*p*=0.002), vertical length of the ears (*p*=0.002) and conchae height (mm) (*p*<0.001).

In the control group, only the mean facial height (mm) was higher in females (*p*=0.032), and in the case group, the nasal root depth (mm) was significantly higher in males (*p*=0.026). The remaining measurements did not differ between the sexes (Table 1).

Of the 18 craniofacial photoanthropometric indices analyzed, only CWxCL (%) (conchae width/length) was statistically higher in the patients in the case group (*p*=0.003) (Table 2).

**Table 1:**
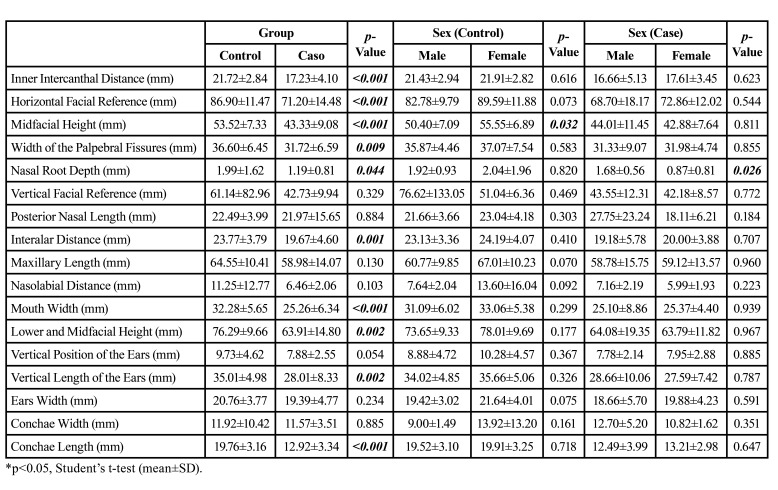
Characterization of individual photoanthropometric measurements used to assess percentage distribution (based on the methodology of Stengel-Rutkowski *et al*., 1984) sorted by syndromic and nonsyndromic diagnosis for OI and by sex.

**Table 2:**
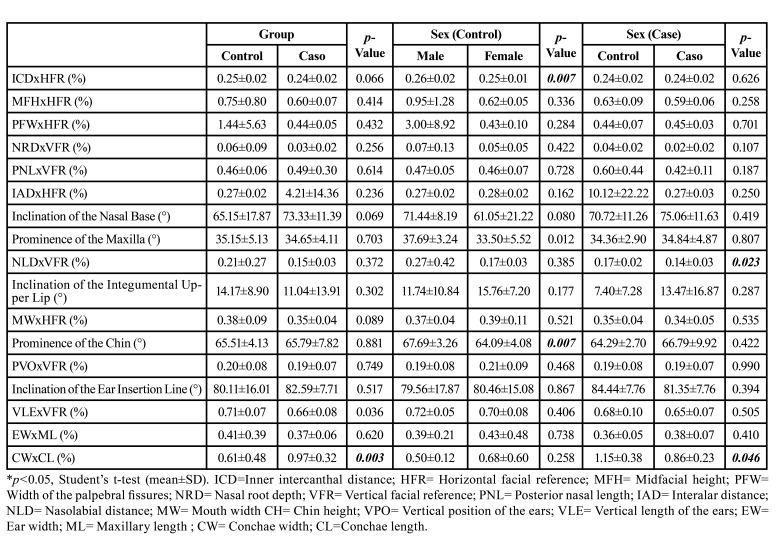
Evaluation of photoanthropometric parameters based on the methodology by Stengel-Rutkowski *et al*., 1984 sorted by syndromic and nonsyndromic diagnosis for OI and by sex.

Furthermore, ICDxHFR (%) (inner intercanthal distance/horizontal facial reference) (*p*=0.007) and prominence of the chin (°) (*p*=0.007) were statistically higher in males than in females in the control group. NLDxVFR (%) (nasolabial distance/vertical facial reference) (*p*=0.023) and CWxCL (%) (conchae width/length) (*p*=0.046) were also statistically higher in males in the case group (Table 2).

Individual measurements: inner intercanthal distance (mm) (*p*<0.001), horizontal facial reference (mm) (*p*<0.001), midfacial height (mm) (*p*<0.001), width of palpebral fissures (mm) (*p* =0.008), nasal root depth (mm) (*p*=0.038), vertical facial reference (mm) (*p*=0.018), posterior nasal length (mm) (*p*=0.044), interalar distance (mm) (*p*=0.003), nasolabial distance (mm) (*p*=0.004), mouth width (mm) (*p*<0.001), total facial height (mm) (*p*=0.001), vertical length of the ears (mm) (*p*=0.002), and conchae length (mm) (*p*<0.001), in addition to the percentage distributions VELxVFR (%) (vertical ear length/vertical facial reference) (*p*=0.012) and CWxCL (%) (conchae width/length) (*p*<0.001), proved to be significant diagnostic predictors in the case group. The cutoff points, sensitivity, and specificity values ​​are shown in Table 3 and Table 4.

**Table 3:**
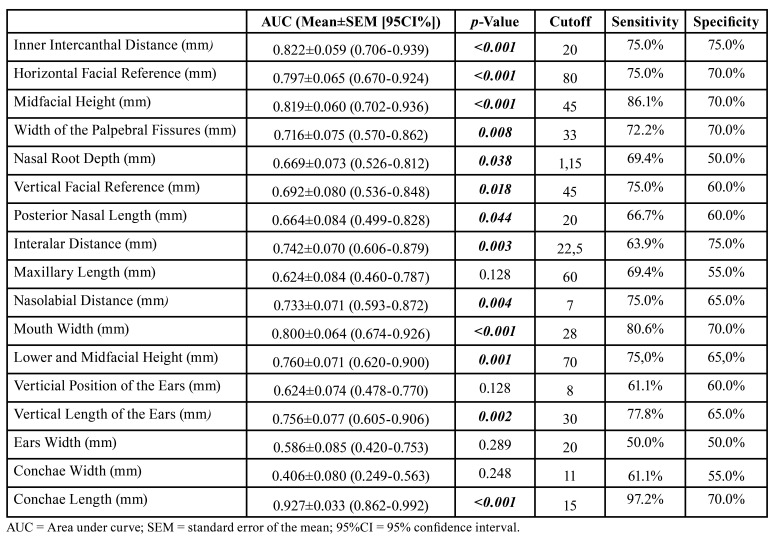
Cutoff points, sensitivity, and specificity values of the individual photoanthropometric distances used to assess percentage distribution (based on the methodology of Stengel-Rutkowski *et al*., 1984).

**Table 4:**
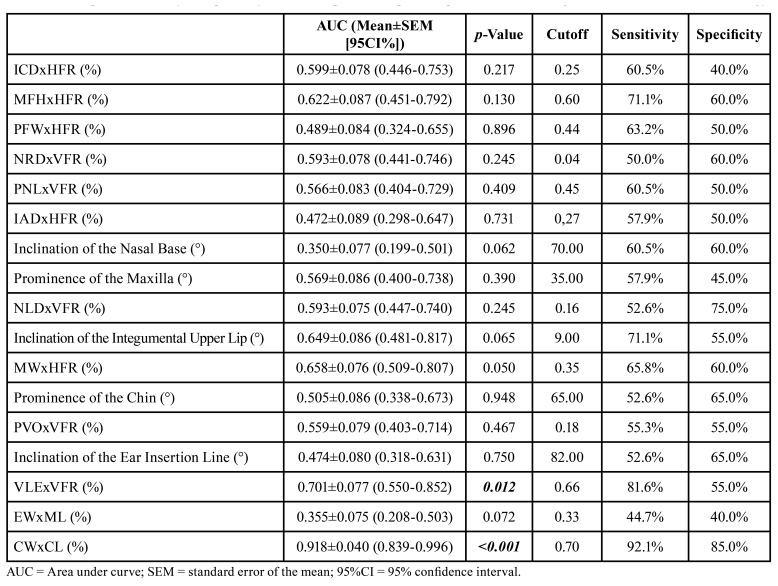
Cutoff points, sensitivity, and specificity values of the photoanthropometric parameters of the Stengel-Rutkowski *et al*., 1984 methodology.

## Discussion

The results of this research evidenced a general reduction in most individual measurements in the photoanthropometric indices of patients with OI, in addition to statistically significant changes in the vertical length of the ears in relation to the vertical facial reference and the conchae width in relation to their length. These findings are relevant for the phenotypic characterization and consequent diagnosis of OI, given the rarity of this condition. A study conducted on a Brazilian population revealed that families of patients with OI commit a significant part of their income to bear the medical and non-medical costs arising from the repercussions of this syndrome on the individual’s life, estimating a loss of income that might exceed 100%, while most of these families did not receive any government assistance benefits (12). The financial burden and the morbidity that this condition brings to the patient highlight the importance of carrying out studies that seek to achieve the early diagnosis of rare diseases, which also supports the discussion on financial assistance and the provision of health services for these individuals.

The craniofacial aspects of OI had not been the main focus of its initial reports, in which bone fragility, blue sclera, and deafness gained greater prominence in the definition and characterization of the syndrome (13). In the 20th century, the description of craniofacial characteristics remained widely subjective and referred to a qualitative evaluation, focusing on the triangular shape of the face and the advanced mandibular position in relation to the anteroposterior position of the maxilla (14,15). Since the implementation of therapeutic protocols (bisphosphonates), which lead to greater bone stability and higher quality of life for these patients, other morphological alterations of the syndrome have gained greater attention in more recent studies, including craniofacial changes (3,16). On the other hand, the characterization approach considering facial parameters from the apparent face (soft tissues) of this syndrome as presented by this investigation is unprecedented in the literature.

Ghoddousi *et al*. (2007) (17) cited three different methods for facial analysis of soft tissues, namely: manual anthropometry analysis, 3D stereophotogrammetry, and 2D photography. Although two-dimensional measurements generate greater distortions than manual anthropometric measurements, they allow for a greater possibility of study analysis and documentation. Three-dimensional stereophotogrammetric measurements were demonstrated to be comparable to manual measurements, albeit resulting in slightly better values. In fact, all three measurement methods exhibited satisfactory levels of reproducibility. Photoanthropometry has been shown to be a viable and reliable option to assess facial parameters using soft tissue references. The methodology proposed by Stengel-Rutkowski *et al*. (1984) (11) has been considered the most adequate methodology for facial assessment in the study of morphological proportions of the face.

Furthermore, this photoanthropometric methodology has been proven effective for the evaluation of other syndromes. Butler *et al*. (1988) (18) found larger dimensions of palpebral fissures and decreased inner canthal distance in patients with fragile X syndrome. Another investigation headed by this author applied the same methodology in a study conducted on patients with Prader-Willi syndrome under hormone therapy compared to patients without hormone therapy (19). Midfacial height, interalar distance, and chin height were increased in patients undergoing hormone therapy; however, as a controversial result, the ears, already mentioned as altered in the literature, were not accentuated in the analysis. Other authors studied patients with Williams syndrome and reported greater midfacial height and width of the palpebral fissure, wide interalar distances, short posterior nasal length, prominent ears with a long and narrow concha, increased chin height, increased ear inclination, and narrow bizygomatic diameter (20). Finally, Gorczyca *et al*. (2012) (21) also applied these same craniofacial parameters in pediatric patients with autism and Asperger's syndrome, frequently observing rotated ears and lengthier posterior nasal base in these patients. Our study was the first to have a control group matched by sex, age, and Legan and Burstone facial analysis.

Matching by Legan and Burstone analysis of the facial aspect, as well as the other variables mentioned above, was another way to eliminate distortions related to a class I, II, or III malocclusion profile that were not inherent in OI. Facial patterns of malocclusion I, II, and III present several intrinsic alterations and well-defined facial changes (10), and matching these parameters was important to establish, for example, the differences between OI patients with Class III malocclusion and control patients with Class III malocclusion. In addition, matching patients by sex and age also minimized the risk of bias because of associated confounding factors.

Comparisons between individual measurements were statistically reduced in patients with OI. However, it should be mentioned that the photographic standardization used did not scale the radiographs to their original size. Most of the analyzed variables did not show a significant difference between the sexes, except for the nasal root depth, the nasolabial distance in relation to the vertical facial reference, and the conchae width in relation to their height, which were statistically higher in males in the case group, indicating homogeneity of the results between males and females. This finding should also be interpreted with caution, as the images were not dimensioned in their original size. It is worth noting that the photographic standardization used in this study was aimed at not distorting facial proportions or creating a barrel effect on the faces of the evaluated individuals, as it follows the original methodology of Stengel-Rutkowski *et al*. (1984) (11).

Most changes in the methodology used were not statistically associated with the OI group. Nevertheless, an important finding was the changes in the ear region in these individuals. The ratio between the width and height of the auricular concha was increased, indicating horizontal flattening (shortened ears). In addition, a greater vertical length of the ears in relation to the face was also observed in individuals with OI. Because OI is a rare condition, the authors of the present investigation believe that these results are very relevant and should be considered in future well-designed case-control studies that include a larger sample of patients.

In previous investigations, subjective facial assessments demonstrated significant facial discrepancies among OI patients; however, this was not observed in the present study. The variability in anatomic characteristics among the syndrome subtypes may explain the lower presence of alterations detected. Therefore, the investigation of these alterations by syndrome subtype might generate different results compared to those of the present research, especially in patients with OI subtypes III and IV, whose phenotypes exhibit greater anatomical and morphological alterations, as observed in the craniofacial cephalometric findings by Waltimo-Sirén *et al*. (2005) (3).

The findings of the present study corroborate the scientific literature regarding the facial changes observed in individuals with OI, while also including parameters related to the ear region analyzed in an unprecedented fashion. Moreover, these data could be potentially considered as additional clinical tools in the diagnostic process of OI. Nonetheless, further studies using photoanthropometric parameters conducted on different populations and ethnic groups are needed.

As a limitation of the present research, we can mention that the data referring to the chin height were not clear in the measurement due to mental inclination with deflection of the submetonian tissues in many patients. For this reason, they were not included for statistical analysis or presented in the results Tables. In addition, as previously described, the population studied came from a single region of northeastern Brazil, belonging to the same ethnic group, which limits the extrapolation of the results to other populations. Because of the rarity of the syndrome and the consequent difficulty in obtaining a sample with a larger number of patients, the present study was limited to analyzing OI without considering its subgroups separately, which infers a need for caution in the interpretation of the results.

Based on the results of this study, we can conclude that narrower but taller ears (in relation to the face) can be considered as alterations inherent in individuals with OI and the patient's sex was not related to changes of the evaluated facial dimensions, except for nasal root depth, nasolabial distance in relation to the vertical facial reference, and the conchae width in relation to their height in males.
